# Comparison between conventional and chemomechanical approaches for the removal of carious dentin: an *in vitro* study

**DOI:** 10.1038/s41598-020-65159-x

**Published:** 2020-05-15

**Authors:** Tito Marcel Lima Santos, Eduardo Bresciani, Felipe de Souza Matos, Samira Esteves Afonso Camargo, Ana Paula Turrioni Hidalgo, Luciana Monti Lima Rivera, Ítalo de Macedo Bernardino, Luiz Renato Paranhos

**Affiliations:** 1 Postgraduate Program in Dentistry, Federal University of Sergipe (UFS), Aracaju, SE Brazil; 2 Department of Restorative Dentistry, Institute of Science and Technology, São Paulo State University (Unesp), São José dos Campos, SP Brazil; 3 Postgraduate Program in Dentistry, School of Dentistry, Federal University of Uberlândia (UFU), Uberlândia, MG Brazil; 4 Department of Restorative Dental Sciences, University of Florida (UF), Gainesville, FL USA; 5 Department of Pediatric Dentistry, School of Dentistry, Federal University of Uberlândia (UFU), Uberlândia, MG Brazil; 6 Department of Dentistry, School of Dentistry, University of Araraquara (UNIARA), Araraquara, SP Brazil; 7 Department of Dentistry, Paraíba State University (UEPB), Campina Grande, PB Brazil; 8 Department of Preventive and Social Dentistry, School of Dentistry, Federal University of Uberlândia (UFU), Uberlândia, MG Brazil

**Keywords:** Dental caries, Dental biomaterials, Minimal intervention dentistry, Restorative dentistry

## Abstract

The present study aimed to evaluate the efficiency, effectiveness, and biocompatibility of two agents used for the chemomechanical removal of carious dentin. Sixty extracted carious human teeth were treated with a conventional bur (CBG) or chemomechanical agents – Papacarie Duo (PG) and Brix 3000 (BG). Treatment efficiency and effectiveness were assessed by the working time for carious dentin removal and Knoop microhardness values, respectively. Human pulp fibroblasts (FP6) were used to evaluate cytotoxicity by incorporating MTT dye, and genotoxicity was evaluated with the micronuclei test. The carious tissue was removed in a shorter time with CBG (median = 54.0 seconds) than the time required for chemomechanical agents (p = 0.0001). However, the time was shorter for Brix 3000 (BG) than that for Papacarie Duo (PG), showing mean values of 85.0 and 110.5 seconds, respectively. Regarding microhardness testing, all approaches tested were effective (p < 0.05). The final mean microhardness values were 48.54 ± 16.31 KHN, 43.23 ± 13.26 KHN, and 47.63 ± 22.40 KHN for PG, BG, and CBG, respectively. PG decreased cell viability compared to that of BG, but it presented no genotoxicity. Brix 3000 may be a good option for chemomechanical dentin caries removal due to its reduced removal time and lower cytotoxicity compared to the other treatment options.

## Introduction

Caries is a multifactorial disease caused by an imbalance in the demineralization and remineralization processes on dental hard tissues, and this imbalance might lead to progressive tooth destruction. This imbalance is preceded by a microbiological shift in biofilm, characterized by an acidogenic and aciduric (cariogenic) population. The shift in the bacterial population is related to the consumption of fermentable dietary carbohydrates. The treatment of the disease depends on the reduction of cariogenic bacteria and the arrest or control of their sequelae (the caries lesion)^1^.

The caries process presents a high prevalence in all age groups^2^. Clinically, the chronic or acute classification of lesions has critical significance because it determines the risk of progression of lesions^3^. The acute caries lesion is more likely to advance, and if no early treatment is performed, it may develop toward the pulp, reaching over 2/3 of the dentin and consequently leading to painful symptomatology, and possibly require endodontic treatment and even tooth extraction^2,4^.

Minimally invasive dentistry (MID) is a philosophy of treating dental caries not only by treating cavities but also by modifying patients’ behavior considering fillings; however, it is not curative^5^. Within this philosophy, when a restoration is needed, the preservation of dental tissue is targeted^6^. Selective removal of carious dentin must be performed^6–8^, considering not only healthy tissue preservation but also the minimization of painful stimuli. The removal of infected dentin and the conservation of affected dentin are based on the carious dentin characteristics of bacterial load and collagen denaturation^9^. The data from clinical studies using this approach reveal no difference in treatment success and reduced chances of pulp exposure^10^, while a more recent report favors the use of selective carious dentin removal in deep lesions, with a probability of success four times higher than the conventional excavation technique^11^.

The clinical dissociation between the infected and affected dentin is not an easy task as this step should follow subjective clinical parameters^7^. Dentin hardness has been advocated as the best parameter for caries removal, and clinicians should assess this characteristic for a proper and effective clinical protocol^6,7,12^. To aid the clinical approach, several excavation protocols are available, and the use of chemomechanical agents is an option, as they act on denatured collagen fibers within the necrotic (infected) dentin layer, preserving the demineralized dentin (affected layer).

The first agents used for chemomechanical removal (based on n-monochloroglycine and sodium hypochlorite) appeared in 1972 but presented low effectiveness and slow action^13,14^. In the 1990s, a new product was introduced in the market (Carisolv), consisting of a gel with two components - one based on 0.5% sodium hypochlorite and the other on amino acids (glutamic acid, leucine, and lysine), sodium chloride, erythrosine, and distilled water. Even though it was considered effective and easy to use, the product was expensive and required customized dentin excavators^13,14^. The literature reports that this approach seems to be similarly efficient to conventional bur excavation regarding bacterial reduction, although there is considerable heterogeneity among the studies, which weakens such an observation^15^.

In 2003, an agent extracted from the papaya peel was presented (Papacarie), and one of its components was papain, which is an enzyme similar to human pepsin. This enzyme breaks denatured collagen fibers, allowing easy removal with hand pieces. The agent is also composed of chloramine, which chemically softens the carious dentin and connects to the degraded collagen portion and toluidine blue with antimicrobial action^16^. Its use presented satisfactory results compared to those of an atraumatic restorative treatment^17^ and those of other chemomechanical removal agents in both permanent^18^ and primary^19^ teeth. In recent systematic reviews and meta-analyses of primary teeth, Papacarie has been shown to be adequate considering the bacterial removal from carious dentin to exert less pain during the procedure, although it requires a longer operating time^20,21^.

More recently, in 2016 (in Latin America), a new papain-based agent (Brix 3000) was introduced to the market, with major composition differences. According to the manufacturer, due to encapsulation and higher concentrations, this product is able to remove the compromised tissue more easily and without causing damage or pulp cytotoxicity. However, studies on this material are scarce in the literature.

Although the biocompatibility of health products is an important characteristic to consider regarding a product, the available data in the literature considering this property of chemomechanical agents is very limited^22^, warranting more studies.

The aim of this study was to evaluate the efficiency and effectiveness of two chemomechanical removal agents compared to that of conventional treatment with rotary instruments by analyzing the working time for carious dentin removal and dentin microhardness tests in an *in vitro* model. Additionally, the biocompatibility of the two papain-based products (Brix 3000 and Papacarie Duo) was assessed in human pulp fibroblasts (FP6) by MTT assays (cytotoxicity) and micronuclei tests (genotoxicity). The authors tested the following hypotheses: (1) there is no difference in dentin microhardness between chemomechanical agents and conventional caries removal, (2) there is no difference in working time for carious tissue removal between chemomechanical agents and conventional caries removal, and the chemomechanical agents tested present (3) no cytotoxicity and (4) no genotoxicity.

## Materials and Methods

To properly present the methodology, it was divided into two sections: mechanical testing and biocompatibility assessment. The methodology and the results presented in this work are part of the Master’s Thesis of the first author (T.M.L. Santos), which is available at the Federal University of Sergipe’s digital library of Theses and Dissertations: https://ri.ufs.br/handle/riufs/13164.

The CRIS (Checklist for Reporting *In vitro* Studies) tool^23^ was used for designing and writing the results according to the recommendations for *in vitro* studies.

### Mechanical testing

#### Study design and sample size

This is an *in vitro* experimental study of extracted permanent human molars with carious dentin. The teeth were donated to dental offices after a dentist confirmed the need for extraction. Considering that extracted human teeth were used, all guidelines related to research ethics involving human beings were respected, and the protocol was approved by the Research Ethics Committee of the Federal University of Sergipe (Certificate of Presentation for Ethical Consideration: 71551417.4.0000.5546). All patients involved in the study provided informed consent for tooth donation for research purposes before tooth extraction and donation.

The sample size calculation was based on the experimental design and the main outcome of the study (continuous quantitative KHN - Knoop hardness number) using the following parameters: two-tailed 5% significance level (α = 0.05), 95% confidence interval, 90% statistical power (β = 0.10), 1:1 ratio of specimen allocation in the experimental groups, and large estimated effect size (d = 0.80), which indicated the need to include a minimum of 20 specimens in each group^24^. For the effect size, data from the pilot study were used for this calculation, resulting in an effect size of 0.8080 (the mean values were 16.2, 26.7, and 29.9 for CBG, PG and BG, respectively, and the SD within groups was 12). A total of 36 readings per group were performed in the pilot study.

Therefore, the final study sample of 60 specimens is suitable and meets the requirements. The sample size was calculated with G*Power software (version 3.1). The sample size was calculated based on the primary outcome of the study, which is the effectiveness of the chemomechanical protocols tested according to the Knoop hardness assessment.

#### Selection and preparation of the samples

The teeth were selected according to the following inclusion criteria: extracted permanent molars with occlusal carious lesions (class I) and the presence of deep or very deep dentin carious lesions, compromising at least 2/3 of the dentin mesiodistally, buccolingually and occlusocervically (confirmed clinically and radiographically). The exclusion criteria were as follows: the presence of sclerotic dentin (determined by clinical hard consistency with a dental explorer and very well polished dentine with a glossy appearance)^25,26^, the presence of a previous restoration covering part of the lesion, and the presence of pulp exposure. In case of doubt, especially considering the subjectivity for determining the sclerotic dentin, the teeth were excluded. Excluded teeth were eliminated through a proper discard process for biological material. After teeth (n = 60) were selected according to the inclusion and exclusion criteria, they were randomly divided into three groups (n = 20 per group) considering the tested approach for carious dentin removal: conventional bur treatment (“gold standard” - CBG), Papacarie Duo (PG), and Brix 3000 (BG). The groups were allocated randomly, considering that all teeth were collected prior to analysis.

The teeth selected were maintained in a refrigerated (8–10 °C) saline solution, which was replaced every week, and the teeth were used within three months.

The teeth included in the study were mounted in self-curing acrylic resin blocks (Dencor, São Paulo, SP, Brazil) with the roots inside the blocks and crowns completely outside. Then, the teeth had their crowns sectioned mesiodistally in the center of the carious lesion into two equal-sized carious lesions, followed by root removal. Both steps were performed with the help of a diamond disc (KG-Sorensen, Cotia, SP, Brazil). The cutting procedures were performed in Labcut machines (Mod.1010 Extec, Enfield, Hartford, CT, USA) and with a Neuone high rotation lathe (F5 - 20000 RPM, São Paulo, SP, Brazil). The crown hemisections were stored in distilled water at room temperature. The surfaces were flattened in the Panambra polisher (Mod. DP10, São Paulo, SP, Brazil) with 80-, 600-, 800-, and 1200-grit polishing paper discs.

#### Caries removal procedure

One half of each tooth was subjected only to the initial microhardness test to obtain the microhardness scores of the dentin regions not subjected to carious tissue removal. The other half of each tooth was subjected to the respective tested approach for carious dentin removal. Caries excavation for PG and BG followed their respective manufacturer’s instructions. The dentin caries were covered with Papacarie Duo gel for 40 seconds (PG) or with Brix 3000 gel for 2 minutes (BG), and the carious dentin was gently scraped away with a blunt dentin spoon in pendulum movements and without pressure to remove the softened carious tissue. Finally, the gels were removed with a water-soaked cotton pellet. In both the PG and BG groups, the gels were applied once, and the excavation was performed until complete caries removal was confirmed by the tactile method of caries detection^27^. CBG was subjected to carious dentin removal using a low-speed round carbide bur #5 in circular scratching movements from the occlusal to the cavity floor until reaching a hard consistency of the dentin by probing. The entire experiment was performed by a single operator who was previously trained on the employed excavation methods. The operator was trained in two pilot studies by an experienced operator on minimally invasive protocols for carious dentin removal (EB), considering the clinical characteristics targeted for the affected dentin. For this training, only dentin hardness was considered, as it is reported to be the only clinical parameter for demineralized and remineralizable carious dentin. The target used in the present study was the leathery/firm dentin as described in a consensus for carious dentin removal^8^.

#### Knoop microhardness testing (effectiveness – primary outcome)

Dental microhardness was analyzed with a microhardness tester (Model FM-700 Future-Tech, Tokyo, Japan) and Knoop indenter. For this test, crown hemisections were inserted in acrylic resin so that the surface evaluated was parallel to the base of the resin. One of the hemisections of each crown not subjected to carious tissue removal was subjected to microhardness analysis to obtain the initial microhardness scores of the dentin regions, while the other hemisection was assessed for microhardness after carious dentin removal to obtain the scores of the dentin regions subjected to carious tissue removal.

Printed photographs were used to aid in delimiting the areas to be tested. Indentation parameters of 25 grams for the static load and a time of 5 seconds were used in this study. Dentin was assessed in three vertical lines within the lesion from the pulp floor toward the pulp. Four indentations in each vertical line were performed (ISO 10993-5) at a distance of 50 µm between the indentations, resulting in indentation at the cavity floor and 50 µm, 100 µm, and 150 µm from the cavity floor. The calculated mean of these indentations at each depth represented the values obtained at the four assessed depths.

#### Analysis of operating time for carious dentin removal (efficiency)

Efficiency was analyzed by the time spent in the procedure and assessed with a digital chronometer (Vollo - VL1809, Curitiba, PR, Brazil) with units of 1/10 seconds. Time was counted from the beginning to the complete removal of the infected dentin, with a low-speed #5 round bur or a blunt dentin spoon associated with the chemomechanical agents tested.

### Biocompatibility assessment

#### Group planning and preparation of extracts

Papacarie Duo and Brix 3000 were organized into two groups for the *in vitro* cytotoxicity and genotoxicity tests with human pulp fibroblasts (FP6) provided by the cell bank of the Laboratory of Cell Biology of the São Paulo State University (UNESP), Campus São José dos Campos, SP, Brazil. The entire research method was performed according to the guidelines of the International Organization of Standardization (ISO) 10993-5:2009 in duplicate.

The indirect contact test for the sensitization of fibroblastic cells was performed with extracts of both products tested, produced from the diffusion of the components from each product to the culture medium, and put in contact with the fibroblastic cells. Therefore, 0.2 grams of each material was placed at the bottom of the 24-well plate (Prolab, São Paulo, SP, Brazil) and covered with 1 ml of DMEM (Dulbecco’s Modified Eagle Medium – LGC Biotecnologia, Cotia, SP, Brazil). The plates were incubated in the dark for 24 h at 37 °C to form the original extracts (1:1) of each material, which were then serially diluted (1:2, 1:4, 1:8, 1:16, and 1:32) prior to testing.

### Cytotoxicity test

Human pulp fibroblasts (FP6) were cultivated in DMEM supplemented with 10% fetal bovine serum (FBS) (Invitrogen, New York, USA) and 1% penicillin/streptomycin (Sigma-Aldrich, St. Louis, MO, USA) at 37 °C and 5% CO_2_, up to 85% confluence. Then, 8,000 cells were placed in each well of 96-well plates (Prolab, São Paulo, SP, Brazil) and incubated for 24 h at 37 °C. After this period, the old medium was removed, and the cell cultures were exposed to the original extracts (1:1), the dilutions of each material (1:2, 1:4, 1:8, 1:16, and 1:32), and 200 μL of the culture medium for the control group, and incubated for 24 h at 37 °C (5% CO_2_). Discarding the exposure medium stopped the exposure of cell cultures after 24 h. Then, the plate was washed three times with 200 μL of sterile PBS to discard dead cells and residue. After washing, 100 μL of the MTT reagent (3-(4,5 dimethylthiazol-2yl)-2,5-diphenyltetrazolium bromide) (Life Technologies, Carlsbad, USA) was added to 0.5 mg/ml in each well. The plates were covered with aluminum foil and incubated for 1 h at 37 °C (5% CO_2_). Then, the MTT solution was removed, and 100 μL of DMSO (99.9%) (dimethyl sulfoxide - Sigma Aldrich Co., Germany) was added for 10 minutes to solubilize the content of the wells. The plates returned to incubation for 10 minutes, followed by placing the plate in the orbital shaker for 10 additional minutes. The absorbance of the wells was read in a microplate spectrophotometer (Cambrex ELx808cse) at 570-nm wavelength, and the data were obtained by Gen5 Data Analysis Software (BioTek U.S. - World Headquarters, USA).

Cytotoxicity was expressed as a percentage relative to the control group (= 100%).

### Genotoxicity test

Genotoxicity was evaluated with the micronuclei test (FluoroShield with DAPI) to detect some forms of chromosome mutations. For this detection, 3 × 10^5^ human pulp fibroblasts (FP6) were cultivated with 1 mL of DMEM supplemented with 10% FBS in 24-well plates (Prolab, São Paulo, SP, Brazil) for 24 h at 37 °C in an atmosphere of 5% CO_2_. The cells were exposed to 1:8 and 1:16 dilutions of Brix 3000 and Papacarie Duo for 24 h. Additionally, there was a negative control (cells only) and a positive control with 5 mM EMS (Sigma-Aldrich, Brazil). Then, the supernatants were discarded, and two washes were performed with buffered saline solution (free from calcium and magnesium, CMF-PBS) to remove nonviable cells. Later, the cells were fixed with 4% formaldehyde for 10 minutes. After additional washing, 200 µL of PBS and a drop of FluoroShield with DAPI were added. The plate was agitated on an orbital table (Solab, Piracicaba, SP, Brazil) for 5 minutes under light protection. The plates were analyzed under a fluorescence microscope (Axiovert 200, Zeiss, Jena, Germany), and the micronuclei were counted every 2,000 cells.

### Statistical analysis

For hardness, nonparametric tests were used to determine the significance of intergroup and intragroup differences, considering that the data were not normally distributed (Shapiro-Wilk test)^28^. The Wilcoxon signed-rank test was used to determine significant intragroup differences in relation to the microhardness scores of the region subjected to carious tissue removal and the region not subjected to it. An indication of the magnitude of statistical variation was evaluated by estimating the effect size (ES)^29,30^. The ES statistics were calculated by dividing the mean microhardness score change by the standard deviation (SD) of the scores observed in the untreated region. The ES values were classified as follows^31^: ≤0.2 indicated a small effect, 0.3–0.7 indicated a medium effect, and ≥0.8 indicated a large effect. Last, the Kruskal-Wallis test was applied to identify significant intergroup differences (PG vs BG vs CBG). All analyses were performed with IBM SPSS Statistics software with a 5% significance level (p < 0.05).

For the operating time, the data were analyzed with IBM SPSS Statistics software (SPSS for Windows, Version 20.0, Armonk, NY, IBM Corp.). The assumption of data normality was not confirmed after applying the Shapiro-Wilk test, and the Kruskal-Wallis test was applied to identify significant intergroup differences at p < 0.05 (PG vs BG vs CBG)^28^.

For cytotoxicity, the data presented a normal distribution (Shapiro-Wilk test), and the means of each product tested at each dilution were statistically analyzed by ANOVA and complemented by Tukey’s test, at 5% significance (p < 0.05)^28^, with the statistical software GraphPad Prism 6.0 (GraphPad Software Inc., San Diego, CA, USA).

For genotoxicity, as the data were normally distributed (Shapiro-Wilk test), they were statistically analyzed by ANOVA and complemented by Tukey’s test, at 5% significance (p < 0.05)^28^, with the statistical software GraphPad Prism 6.0.

## Results

### Knoop microhardness testing (effectiveness)

Table 1 shows the measures of central tendency and variability of longitudinal microhardness scores according to the groups. The intragroup analysis showed significant differences in the mean/median microhardness values of the regions subjected and not subjected to carious tissue removal for the PG (p < 0.001), BG (p < 0.001), and CBG (p < 0.001) groups. After carious dentin excavation, microhardness means of 48.54 KHN (SD = 16.31), 43.23 KHN (SD = 13.26), and 47.63 KHN (SD = 22.40) was found for the PG, BG, and CBG groups. The intergroup analysis did not present significant differences, as Table 2 shows (p > 0.05). As hypothesized, the analysis of changes in magnitude of microhardness showed a large effect size for the CBG (ES = 1.36). Interestingly, the minimally invasive therapies for carious tissue removal also presented large effect sizes of over 0.80 (ES = 1.17 for BG and ES = 1.02 for PG), indicating that these are useful and promising strategies.

**Table 1:** Measures of central tendency and the variability of longitudinal microhardness scores according to the specimen groups subjected to the different strategies for carious tissue removal.

Group	Longitudinal microhardness	Region subjected to carious tissue removal*			Region not subjected to carious tissue removal**		
		Mean (SD)	Median	IQR	Mean (SD)	Median	IQR
**Evaluation**							
PG^(A)^	Interface	40.68 (13.01)	41.27	29.51–51.31	23.98 (11.71)	20.43	15.68–32.38
	50 m	49.21 (15.64)	50.90	34.26–62.53	29.82 (14.00)	28.19	16.70–43.11
	100 m	51.34 (17.42)	50.31	34.38–65.24	36.19 (16.17)	35.15	21.55–50.93
	150 m	52.95 (17.17)	56.68	37.47–65.56	39.65 (16.82)	36.44	23.75–54.76
	**Total**	**48.54 (16.31)**	**49.67**	**34.23–61.01**	**32.41 (15.74)**	**28.69**	**20.13–45.70**
BG^(B)^	Interface	36.12 (13.40)	34.61	29.41–46.26	22.11 (11.64)	15.83	11.65–35.57
	50 m	41.17 (12.03)	43.21	33.38–49.45	24.80 (13.24)	18.57	15.59–36.59
	100 m	46.04 (12.23)	48.73	41.37–55.38	28.85 (13.77)	27.06	16.59–42.28
	150 m	49.60 (12.19)	54.32	44.36–58.83	33.04 (14.53)	32.37	17.76–42.22
	**Total**	**43.23 (13.26)**	**44.64**	**33.38–53.86**	**27.20 (13.73)**	**22.99**	**15.92–38.37**
CBG^(C)^	Interface	38.60 (11.18)	37.94	28.60–46.51	21.24 (11.38)	18.20	10.59–33.27
	50 m	43.84 (12.17)	46.29	30.03–49.75	27.21 (13.51)	28.38	15.02–39.05
	100 m	48.29 (12.56)	47.01	39.53–57.43	30.62 (14.01)	32.24	19.64–43.78
	150 m	59.81 (37.38)	50.89	47.09–61.07	34.73 (14.58)	38.14	20.95–47.04
	**Total**	**47.63 (22.40)**	**46.49**	**36.55–54.39**	**28.45 (14.07)**	**30.29**	**16.23–41.23**

*Note*. PG = Papacarie Duo; BG = Brix 3000; CBG = conventional bur treatment; SD = standard deviation; IQR = interquartile range (25–75 percentile).

^(A)^A significant difference in the microhardness scores of the regions subjected/not subjected to the treatment was observed for PPC (Wilcoxon signed-rank test, p < 0.001).

^(B)^A significant difference in the microhardness scores of the regions subjected/not subjected to the treatment was observed for BRI (Wilcoxon signed-rank test, p < 0.001).

^(C)^A significant difference in the microhardness scores of the regions subjected/not subjected to the treatment was observed for CBT (Wilcoxon signed-rank test, p < 0.001).

*No significant difference was found among the PPC, BRI, and CBT groups when comparing the microhardness scores in the regions subjected to the treatment (Kruskal-Wallis test, p > 0.05).

**No significant difference was found among the PPC, BRI, and CBT groups when comparing the microhardness scores in the regions not subjected to the treatment (Kruskal-Wallis test, p > 0.05).

**Table 2:** Mean differences in microhardness scores using minimally invasive therapies for carious tissue removal, conventional bur treatment, and estimates of effect size (ES).

Group	Region of longitudinal measuring	Mean score change (SD)	Cohen’s d^(a)^
PG	Interface	16.69 (11.73)	1.43
	50 m	19.39 (11.07)	1.39
	100 m	15.15 (13.80)	0.94
	150 m	13.30 (14.03)	0.79
	**Total**	**16.13 (12.68)**	**1.02**
BG	Interface	14.00 (14.71)	1.20
	50 m	16.37 (14.18)	1.24
	100 m	17.19 (16.71)	1.25
	150 m	16.56 (17.63)	1.14
	**Total**	**16.03 (15.62)**	**1.17**
CBG	Interface	17.36 (12.04)	1.53
	50 m	16.62 (14.86)	1.23
	100 m	17.67 (14.07)	1.26
	150 m	25.08 (40.81)	1.72
	**Total**	**19.18 (23.41)**	**1.36**

*Note*. PG = Papacarie Duo; BG = Brix 3000; CBG = conventional bur treatment. SD = standard deviation; (a) effect size [ES statistics were calculated by dividing the mean change of microhardness scores by the standard deviation (SD) of the scores observed in the region not subjected to carious tissue removal].

### Analysis of operating time for carious dentin removal (efficiency)

**Figure 1:**
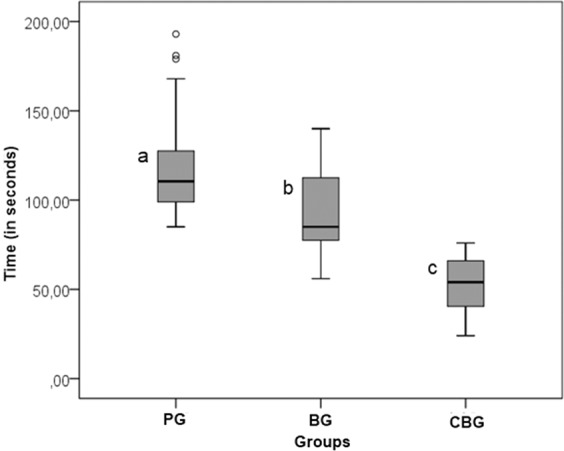
Box plot illustrating the differences in time spent (in seconds) for carious tissue removal using different strategies. PPC = Papacarie Duo; BRI = Brix 3000; CBT = conventional bur treatment. Different letters represent statistically significant differences (Kruskal-Wallis test, p < 0.05). This figure has been published within the Master’s Thesis of the first author (T.M.L. Santos), which is available at the Federal University of Sergipe’s digital library of Theses and Dissertations: https://ri.ufs.br/handle/riufs/13164.

Figure 1 presents the operating time results for carious tissue removal. Significant intergroup differences were observed regarding the time spent on carious tissue removal (p < 0.05), which was the fastest for CBG (median = 54.0 seconds). Regarding the minimally invasive removal therapies, BG (median = 85.0 seconds) performed better than PG (median = 110.5 seconds).

### Cytotoxicity test

**Figure 2:**
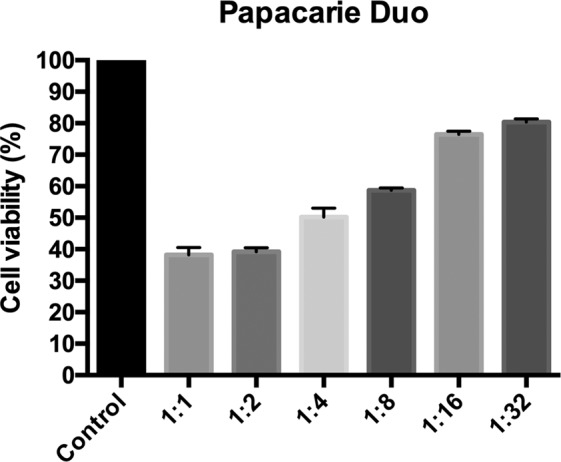
Percentage of cell viability relative to the mean of the control group in different dilutions of Papacarie Duo. Data are expressed as the mean ± the mean standard deviation. Different letters represent statistically significant differences among dilutions of the same material (ANOVA followed by Tukey’s test, p < 0.05). *Indicates a significant difference between the two materials at the same dilution. This figure has been adapted from the Master’s Thesis of the first author (T.M.L. Santos), which is available at the Federal University of Sergipe’s digital library of Theses and Dissertations: https://ri.ufs.br/handle/riufs/13164.

**Figure 3:**
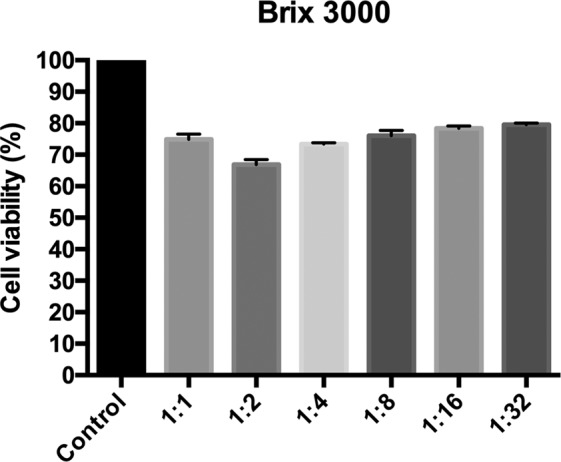
Percentage of cell viability relative to the mean of the control group in different dilutions of Brix 3000. Data are expressed as the mean ± the mean standard deviation. Different letters represent statistically significant differences among dilutions of the same material (ANOVA followed by Tukey’s test, p < 0.05). *Indicates a significant difference between the two materials at the same dilution. This figure has been adapted from the Master’s Thesis of the first author (T.M.L. Santos), which is available at the Federal University of Sergipe’s digital library of Theses and Dissertations: https://ri.ufs.br/handle/riufs/13164.

The results of cell viability (Fig. 2) showed that the original extract (1:1) and the dilutions from 1:2 to 1:4 of Papacarie Duo were cytotoxic, with significant differences compared to the results of the control group (p < 0.05). On the other hand, Brix 3000 (Fig. 3) showed cell viability higher than 60% for the original extract (1:1) and the dilutions tested (1:2, 1:4, 1:8, 1:16, and 1:32), showing no cytotoxicity. Additionally, it was verified that the cell viability of the original extract (1:1) and the dilutions tested (1:2, 1:4, 1:8) of Papacarie Duo were significantly lower than that of Brix 3000 (p < 0.05) (Fig. 2 and 3).

### Genotoxicity test

As expected, the positive EMS control group presented a significantly higher ability to form micronuclei than all the groups tested (p < 0.05). It was verified that 1:8 and 1:16 dilutions of Papacarie Duo and Brix 3000 presented micronuclei formations similar to those of the negative control group and were not considered genotoxic (Table 3) (p > 0.05).

**Table 3:** Mean number of micronuclei found in 2,000 cells.

Group	Micronuclei mean
Papacarie Duo (1:8)	11^a^
Papacarie Duo (1:16)	10^a^
Brix 3000 (1:8)	9^a^
Brix 3000 (1:16)	8^a^
Positive control (EMS)	56^b^
Negative control (Cells only)	7^a^

Different letters indicate statistically significant differences (ANOVA followed by Tukey’s test, p < 0.05).

### Post hoc power analysis

The post hoc power analysis observed in the comparison of the results between Papacarie Duo and Brix 3000 regarding the evaluated secondary outcomes revealed the following results: operating time in seconds (Cohen’s d = 0.98; power = 85.5%), genotoxicity 1:18 (Cohen’s d = 1.47; power = 99.4%) and 1:16 (Cohen’s d = 1.47; power = 99.4%); cytotoxicity 1:1 (Cohen’s d = 15.80; 99.9%), 1:2 (Cohen’s d = 17.45; power = 99.9%), 1:4 (Cohen’s d = 7.82; power = 99.9%), 1:8 (Cohen’s d = 7.82; power = 99.9%), 1:16 (Cohen’s d = 1.22; power = 96.4%) and 1:32 (Cohen’s d = 0.71; power = 59.0%). In general, these findings indicate that the sample size was sufficient to generate statistically reliable results, with large effect sizes (Cohen’s d ≥ 0.80) and statistical power greater than 80% for almost all comparisons.

## Discussion

The present study compared the *in vitro* efficiency (time for carious tissue removal) and effectiveness (dentin microhardness after carious tissue removal) between conventional bur treatment with a rotary instrument and two chemomechanical removal agents. Additionally, the cytotoxicity and genotoxicity of the chemomechanical removal agents Brix 3000 and Papacarie Duo were evaluated. Hypothesis (1) was not rejected, as there was no difference in dentin microhardness between the chemomechanical agents and conventional caries removal.

Restorative treatments with high-speed and low-speed instruments may not preserve healthy tissue^27^. Thus, chemomechanical removal is a good alternative for this principle. The high proteolytic activity of papain, by its action in the denatured collagen molecules, may facilitate the removal of infected dentin, aided by a blunt dentin spoon^13,17,27^. Another great advantage of using these chemomechanical agents is the presence of antiprotease alpha-1-antitrypsin in healthy tissues, which prevents the proteolytic activity of the material. Therefore, only the tissue with denatured collagen fibers is removed (the infected dentin, in this case), and the affected dentin, which is capable of regeneration, is preserved. This preservation shows the importance of using blunt hand instruments to prevent lesions in the healthy tissue^16^.

To evaluate the effectiveness parameters, the cross-sectional microhardness test was performed, in which the intragroup parameter comparison showed that all carious tissue was removed, but there was no statistically significant difference in microhardness among the groups after carious tissue removal. Thus, any of the three options might be recommended for the success of restorative treatment from the carious tissue removal standpoint. It is important to highlight that the present results are based on *in vitro* observations, and clinical investigations should be performed to determine a clinical protocol more accurately.

Similar to the present study, it has been shown that the papain-based agent (Papacarie Duo) was as effective as the sodium hypochlorite-based agent (Carisolv), and both were effective for carious tissue removal^27^. On the other hand, there is evidence that the chemomechanical method results in dentin with hardness values similar to those of sound dentin and higher than those of the conventional mechanical method^32^. The evaluation of effectiveness is rather important from a clinical standpoint because maintaining infected dentin, especially in the external walls, promotes adhesion failures of the restorative material, so only the affected dentin should be maintained in the internal walls^6^.

When analyzing efficiency by the removal time with both materials, a shorter time was observed in the BG group in comparison to that in the PG group, indicating that hypothesis (2) should be rejected. Although the sample size was not calculated for this specific parameter (surrogate outcome), the power after the data were collected was greater than 80%, granting the statistical inference as presented. This outcome may have occurred because of the bioencapsulation of the material, which potentiates enzyme action and is a good alternative for rapid carious tissue removal. This result of a shorter time of the clinical session is rather viable, especially for child patients with difficult dental treatment conditioning. On the other hand, scientific evidence^17^ showed that Papacarie Duo presents a longer removal time than atraumatic restorative treatment (ART). The data of the present study corroborate the findings of the literature regarding the comparison between conventional mechanical methods and chemomechanical methods, with the latter taking longer to reach the expected results^33^. However, because this is the first study to test this property of Brix 3000 and considering it is an *in vitro* investigation, further studies are encouraged to confirm the results. Despite this finding, the time required for the protocols would not negatively affect the clinical activity regarding their application. It is even preferable to report findings showing no need for anesthesia and reduction of sensitivity to the procedure.

The question regarding the sample size according to the efficiency outcome should be pointed out because the size was determined by the primary outcome (hardness assessment). The fact that differences were detected is a good sign, but the lack of data normality reinforces the need for further investigations considering this parameter as the primary outcome.

Hypothesis (3) was rejected, as the present study showed that BG presented lower cytotoxicity than PG, considering its original extract (1:1), and all its dilutions presented high cell viability. From a clinical standpoint, the evaluation of cytotoxicity is important as a guide to show that cytotoxic caries-removing substances should be used carefully due to their potential to cause painful postoperative symptomatology and to develop pulp necrosis, especially in deep carious lesions. However, PG was only cytotoxic up to the 1:4 dilution, showing that this material decreased cell viability at higher concentrations. Our results agree with findings that Papacarie Duo presented slight cytotoxicity after 30 minutes in contact with oral cells (gingival fibroblasts and pulp fibroblasts), suggesting the importance of being cautious when applying this material^34^. In contrast, other evidence^16^ reported that Papacarie Duo was not cytotoxic for pulp fibroblasts.

Regarding genotoxicity, this study found that none of the materials tested (Brix 3000 or Papacarie Duo) were genotoxic because they could not induce high micronuclei formation on pulp cells; hence, hypothesis (4) should not be rejected. Corroborating our results, other observations showed biocompatibility *in vitro* and *in vivo* of papain-based caries-removing substances^22^. Thus, we suggest that Brix 3000 is a good option for clinical caries removal.

Chemomechanical caries removal is a great advantage for the compliance of both children and adults with dental treatment, considering that when anxiety is enhanced by the painful stimulus and the use of anesthesia, it compromises the treatment cooperation of adult patients as well, later risking their quality of life in case the procedure is not finished^35^.

Considering the best performance of BG regarding the operating time and the low cytotoxicity potential, it seems appropriate to extrapolate the present results and point out clinical scenarios in which this material should be indicated. Based on the present data, conditions requiring faster procedures that are potentially closer to the pulp tissue would benefit by employing BG material. The restorative approach in primary teeth, in which faster procedures are targeted and small cavities might be close to the pulp tissue due to reduced tooth dimensions in comparison to permanent teeth, grants that indication. It is worth noting that the primary outcome did not reveal differences and, as the study was designed based on such an outcome, the aforementioned indication might be interpreted with caution.

Similar to other studies, the present study is not free from limitations. The main limitation relates to the laboratory character of the experiment, which restricts extrapolating the results for clinical practice. Moreover, the characterization of the dentin according to clinical aspects is very subjective, which may have influenced the selection of teeth (considering the sclerotic dentin) and carious dentin removal, which was based solely on the clinical properties. Further clinical studies are suggested to fill this gap and bring more clarity to the topic. On the other hand, the present study is original, exposes important information, and contributes to developing the scientific literature as the first study to compare Brix 3000 and Papacarie Duo regarding their efficiency, effectiveness, cytotoxicity, and genotoxicity.

The results of this *in vitro* study suggest that the chemomechanical removal agents evaluated were as effective as the conventional bur treatment for carious tissue removal, indicating them as promising and useful strategies for clinical dental practice. The analysis of efficiency showed that Brix 3000 required a shorter time to remove the carious tissue than Papacarie Duo, and although the latter presented higher cytotoxicity than Brix 3000, none of the products tested were genotoxic.

## Conclusion

Brix 3000 (BG) supports the approach of an effective, efficient, and noncytotoxic or genotoxic chemomechanical therapy due to the reduced removal time and lower cytotoxicity when compared to Papacarie Duo (PG) and conventional bur treatment (CBG) outcomes. It may be used for less invasive restorative processes.

## Data Availability

The datasets generated during and/or analyzed during the current study are available from the corresponding author on reasonable request.
